# Abdominal desmoplastic small round cell tumor without extraperitoneal metastases: Is there a benefit for HIPEC after macroscopically complete cytoreductive surgery?

**DOI:** 10.1371/journal.pone.0171639

**Published:** 2017-02-24

**Authors:** C. Honoré, V. Atallah, O. Mir, D. Orbach, G. Ferron, C. LePéchoux, J. B. Delhorme, P. Philippe-Chomette, S. Sarnacki, S. Msika, P. Terrier, O. Glehen, H. Martelli, V. Minard-Colin, F. Bertucci, J. Y. Blay, S. Bonvalot, D. Elias, A. LeCesne, P. Sargos, French Network for Rare Peritoneal Malignancies (RENAPE), French Pediatric Cancer Society (SFCE), French Reference Network in Sarcoma Pathology (RRePS) French Sarcoma Clinical Network (NETSARC)

**Affiliations:** 1 Department of Surgical Oncology, Gustave Roussy Cancer Campus, Villejuif, France; 2 Department of Radiotherapy, Bergonié Institute, Bordeaux, France; 3 Department of Medical Oncology, Gustave Roussy Cancer Campus, Villejuif, France; 4 Department of Pediatric, Adolescent and Young Adult Oncology, Curie Institute, Paris, France; 5 Department of Surgical Oncology, Claudius Régaud Institute, Toulouse, France; 6 Department of Radiotherapy, Gustave Roussy Cancer Campus, Villejuif, France; 7 Department of General and Digestive Surgery, Hautepierre Hospital, Strasbourg University, Strasbourg, France; 8 Department of Pediatric Surgery, Robert Debré Hospital, Paris, France; 9 Department of Pediatric Surgery, Necker Enfants Malades Hospital, Paris Descartes University, Paris, France; 10 of Digestive Surgery, Louis Mourier Hospital, Paris Diderot University, Colombes, France; 11 Department of Pathology, Gustave Roussy Cancer Campus, Villejuif, France; 12 Department of Surgical Oncology, Lyon Civil Hospices, South Lyon University Hospital Center, Lyon, France; 13 Department of Pediatric Surgery, Kremlin-Bicêtre Hospital, Paris 11 University, Kremlin-Bicêtre, France; 14 Department of Pediatric Oncology, Gustave Roussy Cancer Campus, Villejuif, France; 15 Department of Medical Oncology, Paoli-Calmettes Institute, Aix-Marseille University, Marseille, France; 16 Department of Medical Oncology, Leon Bérard Center, Lyon, France; 17 Department of Surgery, Curie Institute, Paris, France; Johns Hopkins University, UNITED STATES

## Abstract

**Background:**

Desmoplastic Small Round Cell Tumor (DSRCT) is a rare disease affecting predominantly children and young adults and for which the benefit of hyperthermic intraperitoneal chemotherapy (HIPEC) after complete cytoreductive surgery (CCRS) remains unknown.

**Methods:**

To identify patients with DSRCT without extraperitoneal metastases (EPM) who underwent CCRS between 1991 and 2015, a retrospective nation-wide survey was conducted by crossing the prospective and retrospective databases of the French Network for Rare Peritoneal Malignancies, French Reference Network in Sarcoma Pathology, French Sarcoma Clinical Network and French Pediatric Cancer Society.

**Results:**

Among the 107 patients with DSRCT, 48 had no EPM and underwent CCRS. The median peritoneal cancer index (PCI) was 9 (range: 2–27). Among these 48 patients, 38 (79%) had pre- and/or postoperative chemotherapy and 23 (48%) postoperative whole abdominopelvic radiotherapy (WAP-RT). Intraperitoneal chemotherapy was administered to 11 patients (23%): two received early postoperative intraperitoneal chemotherapy (EPIC) and nine HIPEC. After a median follow-up of 30 months, the median overall survival (OS) of the entire cohort was 42 months. The 2-y and 5-y OS were 72% and 19%. The 2-y and 5-y disease-free survival (DFS) were 30% and 12%. WAP-RT was the only variable associated with longer peritoneal recurrence-free survival and DFS after CCRS. The influence of HIPEC/EPIC on OS and DFS was not statistically conclusive.

**Conclusion:**

The benefit of HIPEC is still unknown and should be evaluated in a prospective trial. The value of postoperative WAP-RT seems to be confirmed.

## Introduction

Desmoplastic Small Round Cell Tumor (DSRCT) is a rare abdominal disease affecting predominantly children and young adults [1–3]. Fewer than 500 cases have been reported in the literature [2–4]. The estimated incidence is between 0.2 and 0.5 per million per year [5]. The specific translocation t(11:22)(p13;q12), which fuses the ESWR1 gene to the WT1 gene, allows the formal diagnosis [6–8]. DSRCT has an extremely aggressive clinical course [2]. Peritoneal metastases (earlier referred as peritoneal carcinomatosis or sarcomatosis) are nearly always present at diagnosis and synchronous extraperitoneal metastases (EPM) are found in 47% of patients [9]. In a recent analysis, four good prognostic factors were identified: absence of EPM, complete macroscopic resection of the peritoneal disease, postoperative whole abdominopelvic radiotherapy (WAP-RT) and postoperative chemotherapy [9]. No benefit of surgery was found in patients with EPM [9]. In the absence of comparative studies, there is no consensus concerning the optimal locoregional treatment and the value of hyperthemic intraperitoneal chemotherapy (HIPEC) is not determined. The aim of the present study was to analyze nation-wide in France, the treatment of DSRCT without EPM to determine the potential benefit of HIPEC after macroscopically complete surgery.

## Material and methods

### Patient selection

A nation-wide retrospective survey was conducted to identify patients treated for DSRCT in France between January 1991 and January 2015, by crossing the prospective and retrospective databases of the French Network for Rare Peritoneal Malignancies (RENAPE), French Reference Network in Sarcoma Pathology (RRePS), French Sarcoma Clinical Network (NETSARC) and French Pediatric Cancer Society (SFCE). This study was approved by French Network for Rare Peritoneal Malignancies (RENAPE), French Pediatric Cancer Society (SFCE) and French Sarcoma Group (GSF-GETO) institutional board (the GSF-GETO having scientific authority on French Reference Network in Sarcoma Pathology (RRePS) and French Sarcoma Clinical Network (NETSARC)). A list of all institutional board members is available in supporting information files (S1 File). All data were anonymized prior to access and analysis. DSRCT diagnostic suspicion was based on the tumor histological features. Demographic data, diagnostic circumstances, tumor characteristics, treatment variables, intraperitoneal chemotherapy, perioperative radiotherapy and/or chemotherapy and long-term outcome were retrospectively retrieved from the patients’ files. Patients without confirmation of the t(11:22)(p13;q12) translocation in their files were considered with an uncertain diagnosis of DSRCT and were not included in the study. EPM were diagnosed based on radiological findings either with CT scan, MRI or PET-CT. Whenever available, the peritoneal extent of the disease, evaluated peroperatively with the Peritoneal Cancer Index (PCI), was recorded [10]. All patients were retrospectively staged according to the MD Anderson Cancer Center DSRCT staging criteria [11]. The completeness of peritoneal cytoreduction was retrospectively classified according to the Sugarbaker completeness of cytoreduction (CC) score, as follows: CC0, no residual macroscopic disease; CC1, residual nodules smaller than 2.5 mm; CC2, residual nodules between 2.5 mm and 2.5 cm; and CC3, residual nodules greater than 2.5 cm [11]. Surgery was considered macroscopically complete when CC0 or CC1. Surgical complications were retrospectively graded according to the Clavien-Dindo classification [12]. Complications classified as higher than 2 were considered severe. Patient level data are available in supporting information (S2 File).

### Inclusion criteria

Inclusion criteria were: patients treated for DSRCT in France between January 1991 and January 2015, without EPM, after macroscopically complete surgery. Patients with EPM or after incomplete tumor resection are not considered suitable candidates for intraperitoneal treatment and, therefore, were not included in the study. Conversely, patients with abdominal lymph node metastases, which were not considered as distant metastatic disease, were included in the present study.

### Intraperitoneal chemotherapy

Due to the disease rarity, no specific recommendations are available and intraperitoneal chemotherapy, HIPEC or early postoperative intraperitoneal chemotherapy (EPIC) was performed according to the practice of each Centre. Intraperitoneal chemotherapy modalities (drugs, “closed” or “open” HIPEC technique, temperatures and duration) were retrospectively recorded.

### Long-term follow-up

Recurrence was diagnosed based on clinical or radiological findings and was confirmed during a multidisciplinary team meeting. Progressive disease was defined as tumor growth documented by imaging analysis (MRI or CT scan) according to RECIST [13]. Follow-up data included the date of the most recent follow-up, the patient’s status (alive with disease, alive without disease, dead), the site of the initial recurrence and of all the subsequent recurrences.

### Statistical analysis

The cut-off date for survival analyses (censored data) was July 1, 2015. Data were expressed as the mean ± standard error of the mean (SEM), unless otherwise stated. A p-value lower than 0.05 was considered significant. Categorical variables were compared within groups using the Chi-square or Fisher's exact test, when appropriate. The survival analysis was performed using the Kaplan–Meier method and results were compared using the log-rank test. Overall survival (OS) was calculated from the date of diagnosis to the date of death or of the last follow-up. Disease-free survival (DFS) and peritoneal recurrence-free survival were computed from the date of the primary tumor resection to the date of local or distant recurrence. Gender, age, WHO performance status, lymph node metastases, PCI, MD Anderson Cancer Center stage, preoperative chemotherapy, surgery completeness, HIPEC, EPIC, postoperative complications, postoperative chemotherapy and postoperative radiotherapy were included in the univariate analysis to identify variables that could predict recurrence and/or have a prognostic impact. All statistical analyses were performed using the IBM SPSS Statistics software, version 20.0.

## Results

### Demographic data

**Fig 1:**
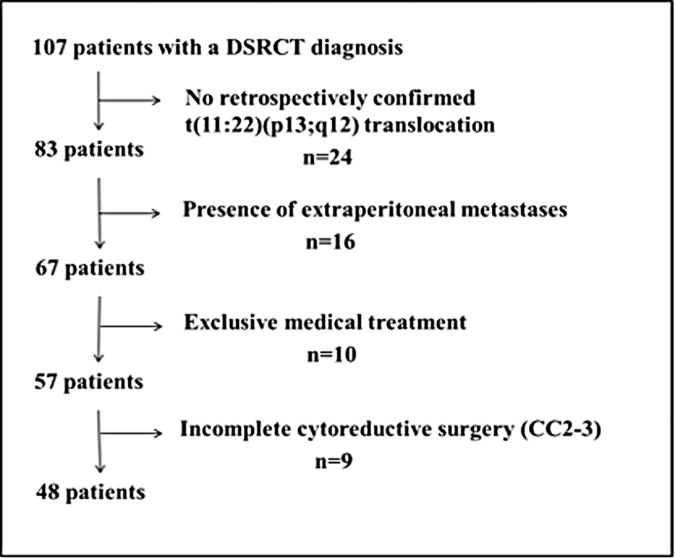
Selection of patients with DSRCT without extra-peritoneal metastases and complete cytoreductive surgery among all the patients with DSRCT.

From the 107 patients identified and treated in eight French centers between 1991 and 2015, 48 patients were selected for the present study (Fig 1). Thirty-five patients were males (73%) and 13 were females (27%). The median age at diagnosis was 22 years (range: 3 to 57). The median PCI at laparotomy, available in 31 patients (65%), was 9 (range: 2 to 27). Fourteen patients (29%) had abdominal lymph node metastases (Table 1).

**Table 1:** Patients and tumors’ characteristics.

Number of patients		48 (100%)
Median age, years [range]		22 [3 – 57]
Gender		
	Male	35 (73%)
	Female	13 (27%)
WHO performance status		
	0	28 (58%)
	1	6 (13%)
	2	1 (2%)
	N/A	13 (27%)
Median PCI [range]		9 [2 – 27]
Lymph node metastases		
	Yes	14 (29%)
	No	34 (71%)
MD Anderson stage		
	I	21 (44%)
	II	10 (21%)
	N/A	17 (35%)

Abbreviations: WHO, World Health Organization; PCI, peritoneal cancer index; N/A, non-available

### Treatment

#### Chemotherapy and radiotherapy

Before surgery, 37 patients (77%) had chemotherapy, mainly a combination of doxorubicin and ifosfamide (24 patients; 50%). Thirty eight patients (79%) had postoperative chemotherapy (more than 15 different regimens were recorded). The combination of doxorubicin and ifosfamide was the most commonly used regimen (11 patients; 23%). Twenty-eight patients (58%) had both pre- and postoperative chemotherapy. No patient had preoperative radiotherapy, whereas 23 patients (48%) received postoperative WAP-RT at the dose of 30 Gy. In four patients, WAP-RT was administered after intraperitoneal chemotherapy.

#### Surgery

All included patients underwent complete CRS. No postoperative in-hospital death was recorded and the postoperative morbidity rate was 17%. The most common severe complication was deep abscess (four patients), followed by digestive anastomotic fistula (one patient), limb compartment syndrome (one patient) and hemoperitoneum (one patient).

#### Intraperitoneal chemotherapy

After complete CRS, 11 patients (23%) had intraperitoneal chemotherapy (Table 2). Two patients received EPIC. Nine patients had HIPEC (open or closed technique) at a temperature between 41°C and 43°c for 30 to 60 minutes. Overall, six patients received cisplatin-based regimes, mainly in combination with mitomycin. Oxaliplatin was the second most commonly used drug, either alone or associated with irinotecan.

**Table 2:** Intraperitoneal chemotherapy.

Type	Drugs	Technique	Duration	Temperature	Doses
HIPEC	Cisplatin + Mitomycin	N/A	N/A	N/A	N/A
HIPEC	Cisplatin + Mitomycin	Open	30 min	42°C	120mg + 75mg/m^2^
HIPEC	Cisplatin + Mitomycin + Irinotecan	Open	N/A	41°C	N/A
HIPEC	Cisplatin	N/A	60 min	41°C	N/A
EPIC	Cisplatin + Adriamycin	Closed	5 days	N/A	15mg/m^2^ + 0.1mg/kg*
EPIC	Cisplatin + Adriamycin	Closed	5 days	N/A	15mg/m^2^ + 0.1mg/kg*
HIPEC	Oxaliplatin	Open	30 min	43°C	N/A
HIPEC	Oxaliplatin	Open	30 min	43°C	460mg/m^2^
HIPEC	Oxaliplatin	Open	30 min	43°C	460mg/m^2^
HIPEC	Oxaliplatin + Irinotecan	Open	30 min	43°C	300mg/m^2^ + 200mg/m^2^
HIPEC	Oxaliplatin + Irinotecan	Open	30 min	43°C	300mg/m^2^ + 200mg/m^2^

Abbreviations: HIPEC, hyperthermic intraperitoneal chemotherapy; EPIC, early postoperative intraperitoneal chemotherapy; N/A, non-available

* daily dose

### Overall survival

**Fig 2:**
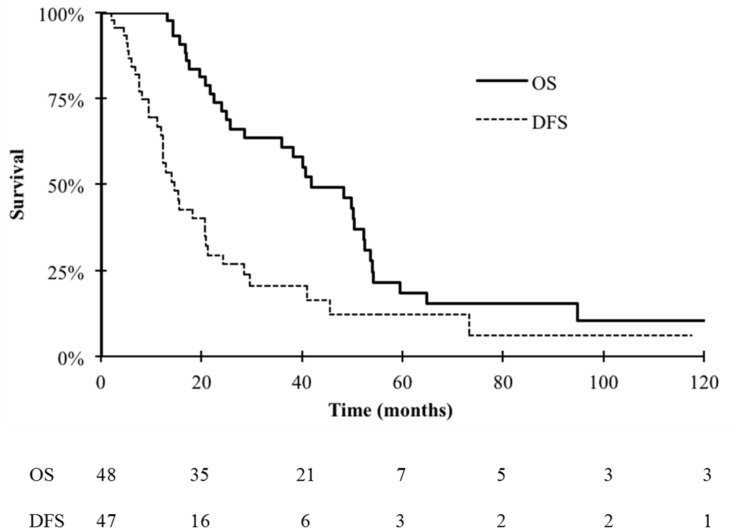
Overall (OS) and disease-free survival (DFS) after complete cytoreductive surgery in patients with DSRCT without extra-peritoneal metastases.

The median follow-up was 30 months (range: 8 to 131 months). Thirty-two patients died during the follow-up period and the specific cancer mortality was 100%. The median overall survival (OS) of the entire cohort was 42 months. The 2-y and 5-y OS rates after complete CRS were 72% (CI 95%: 58–85) and 19% (CI 95% 5–32), respectively (Fig 2). None of the variables (age, gender, lymph node metastases, PCI, MD Anderson stage, preoperative/postoperative chemotherapy, intraperitoneal chemotherapy, WAP-RT, postoperative complications) tested in the univariate analysis was significantly correlated with OS.

### Recurrences

At the cut-off date, 11 patients were alive and disease-free. Five were alive with disease, but only one of them had a follow-up longer than one year after the recurrence, whereas the others were lost to follow-up. The median disease free survival (DFS) was 21 months (range: 5 to 131 months). The 2-y and 5-y DFS rates after complete CRS were 30% (CI 95%: 15–44) and 12% (CI 95% 1–24), respectively (Fig 2). Thirty-seven patients (77%) had a recurrence and the median time to recurrence was 12 months (range: 5 to 73 months). The first recurrence was located in the peritoneum in 23 patients (62%), outside the peritoneum in six patients (16%), or synchronously in and outside the peritoneum in seven patients (19%). The only factor associated with longer DFS was postoperative WAP-RT (p = 0.0014). All other tested variables (age, gender, lymph node metastases, PCI, MD Anderson stage, chemotherapy, IPC and postoperative complications) were not significantly correlated with DFS.

#### Peritoneal recurrences

Overall, 33 patients (69%) had a peritoneal recurrence after a median time of 13 months (range 2–73). The 1-, 2- and 5-year peritoneal recurrence rates were 29% (CI 95%: 16–43), 67% (CI 95%: 52–82) and 93% (CI 95%: 74–99), respectively. Twenty-one percent of peritoneal recurrences occurred within six months after surgery, 48% before one year and 81% before two years. Univariate analysis indicated that intraperitoneal chemotherapy was not predictive of peritoneal recurrence-free survival. The only factor associated with longer peritoneal recurrence-free survival was postoperative WAP-RT (p = 0.0244).

### Influence of HIPEC or EPIC

The median PCI was significantly higher in the HIPEC/EPIC group than in the group without HIPEC/EPIC) (16 vs. 9; p = 0.05). However, OS was not significantly different in these two groups (p = 0.085). The 2-y and 5-y OS rates were 54% (CI 95%: 14–93) and 0% in the HIPEC/EPIC group vs. 74% (IC 95%: 60–89) and 22% (IC 95%: 7–37) in patients without HIPEC/EPIC, respectively. Postoperative morbidity after HIPEC/EPIC was significantly higher (p = 0.05), with a 40% complication rate compared with 10% in patients without HIPEC/EPIC. Severe postoperative complications after HIPEC/EPIC included hemoperitoneum, intra-abdominal abscess and limb compartment syndrome. Nevertheless, they were successfully managed and no mortality was recorded. The 2-y and 5-y DFS rates were 0% and 0% in the HIPEC/EPIC group vs. 34% (CI 95%: 18–51) and 14% (CI 95%: 7 = 1–28) in patients without HIPEC/EPIC, respectively (p = 0.087). Six patients of the HIPEC/EPIC group (55%) had a peritoneal recurrence compared with 35 patients (73%) in the group without HIPEC/EPIC, after a median follow-up of 11 months and 34 months, respectively.

## Discussion

This multicentre retrospective study shows that DSRCT remains a complex disease with a dismal prognosis even when using aggressive multimodal locoregional treatments. After complete CRS, the median OS in patients with DSRCT without EPM was 42 months. No prognostic factor was identified. Moreover, 69% of patients had a peritoneal recurrence after a median time of 13 months. The only factor significantly associated with peritoneal recurrence-free survival and DFS was WAP-RT. Intraperitoneal chemotherapy (HIPEC or EPIC) did not improve survival, although patients who received HIPEC/EPIC had a significantly higher PCI. There were 11 long-term survivors and disease-free up to 131 months after surgery.

### Complete cytoreductive surgery

CRS completeness is a major prognostic factor in patients with DSRCT [9,14,15]. Although, surgeons should anticipate postoperative morbidity and reduction of quality of life linked to the visceral resection required for achieving complete CRS, this should not stop from removing all macroscopic disease. A CC2-3 resection does not bring any survival benefit compared to chemotherapy alone [9]. In our series, the morbidity after complete CRS without HIPEC/EPIC was only 10% and did not prevent postoperative adjuvant treatment in 66% of patients. Invaded abdominal lymph nodes were not a poor prognostic factor and should not be considered as a contraindication to surgery and complete resection of the invaded lymph node(s) should be performed, if technically feasible. Chemotherapy has proved its value in Ewing family sarcoma and aim preoperatively to decrease the tumour bulk [9,14,16–19].

### HIPEC/EPIC

Forty-three patients with DSRCT who received intraperitoneal chemotherapy have been reported in the literature, including our 11 patients, and all after 2010 [15,20–24]. The only randomized controlled trial in patients with peritoneal metastases originating from sarcomas concluded that intraperitoneal chemotherapy (without hyperthermia) did not bring any survival benefit [25]. However, this trial was designed to detect a minimal increase of 40% in OS following the addition of intraperitoneal chemotherapy and was underpowered to demonstrate a smaller difference. No comparative study on DSRCT is available. In a series of 26 patients with DSRCT who received HIPEC after complete CRS, the median OS and DFS rates were 31 months and 9 months, respectively [15], compared with 37 months and 12 months, respectively, in the 37 patients without HIPEC/EPIC of our series. The data analysis on a potential survival benefit with HIPEC or EPIC after complete CRS in patients without EPM suffers from bias that might have obliterated the actual benefit of HIPEC. The first potential explanation might be the small sample size -and thus the lack of statistical power. HIPEC/EPIC benefit could also have been hidden by the effect of another locoregional treatment, for instance radiotherapy. Moreover, as HIPEC/EPIC modalities/drugs were very heterogeneous and potentially ineffective in mesenchymal tumors, the most appropriate intraperitoneal drug might not have been used [21]. Thus far, no intraperitoneal chemotherapy regimen has demonstrated any specific benefit even if the combination of cisplatin and doxorubicin was the only borderline independent determinant of better local control in a retrospective study [21, 26,27]. Even if striking differences remain among treatment protocols, a recent study reported a benefit of HIPEC over EPIC in peritoneal metastases of unusual origin, including DSRCT [28]. Finally, PCI was significantly higher in the HIPEC/EPIC group and this could also have influenced our results, because PCI is a major prognostic factor after surgery in many peritoneal diseases [26,28].

### Whole abdominopelvic radiotherapy

With a local failure rate of nearly 70%, clinicians might want to combine all possible methods to prevent local recurrences. One option is to associate HIPEC with WAP-RT, as done at MD Anderson Cancer Center since 2007 [29]. However, the benefit of this strategy could be mitigated by the potential cumulative late abdominal toxicity. Our findings confirm that postoperative WAP-RT might prevent recurrence and could increase survival [9]. The peritoneal recurrence rate was 47% after WAP-RT and 92% without WAP-RT. Long-term toxicity could hamper the benefit, but the introduction of new radiotherapy techniques reduces the risk of bowel toxicity and recent results show an acceptable rate of late complications [30–32]. In the only study available, seven patients with DSRCT received HIPEC before WAP- intensity-modulated radiation therapy (WAP-IMRT) and none developed grade 3 or 4 toxicity after a median follow-up of 15 months [30]. In our series, four patients had both HIPEC and WAP-RT and none developed grade 2 or higher late toxicity after 14 months of median follow-up.

### Long term adjuvant therapy

With a 5-y DFS of 12% despite an optimal locoregional treatment, a prolonged adjuvant treatment could be evaluated in a prospective trial. The potential candidates are pazopanib, trabectidin, vinorelbine, low-dose cyclophosphamide, trastuzumab, temsirolimus and sunitinib that have all been tested in the metastatic setting with encouraging results and acceptable toxicity [33–40].

The limitation of this study is still the small number of patients treated in multiple centres over a long period and the heterogeneity in terms of HIPEC /EPIC modalities that did not allow providing a high level of evidence. However, by crossing different national databases, this study brings an exhaustive list of patients treated with curative intent since 1991. The next step should be to develop an international, centralized database.

## Conclusions

The benefit of HIPEC or EPIC after complete CRS in patients with a DSRCT without EPM is still unknown. Patients who underwent HIPEC or EPIC in this study likely had more advanced disease with a significantly higher PCI. Conversely, Data suggest a confirmed benefit of postoperative WAP-RT. The benefit of HIPEC should be evaluated in a prospective trial.

## Supporting information

S1 File Institutional Board members of the French Network for Rare Peritoneal Malignancies (RENAPE), the French Pediatric Cancer Society (SFCE), and the French Sarcoma Group (GSF-GETO). (DOCX)

S2 File Patient level data. (XLSX)
