# Supplementary material for: Abdominal desmoplastic small round cell tumor without extraperitoneal metastases: Is there a benefit for HIPEC after macroscopically complete cytoreductive surgery?
Source: PLoS One. 2017 Feb 24;12(2):e0171639. doi: 10.1371/journal.pone.0171639 (PMC5325210; doi:10.1371/journal.pone.0171639)
Supplement: S1 File — (DOCX) [file pone.0171639.s001.docx]

**Members of Institutional Board**

**French Network for Rare Peritoneal Malignancies (RENAPE)**

Frederic BIBEAU (Department of Pathology, Caen University Hospital, Caen, France) [bibeau-f@chu-caen.fr](mailto:bibeau-f@chu-caen.fr)

Dominique ELIAS (Department of Surgery, Gustave Roussy, Villejuif, France) [Dominique.ELIAS@gustaveroussy.fr](mailto:Dominique.ELIAS@gustaveroussy.fr)

François-Noël GILLY (Department of Surgical Oncology, Lyon University Hospital, Lyon, France) [francois.gilly@chu-lyon.fr](mailto:francois.gilly@chu-lyon.fr)

Olivier GLEHEN (Department of Surgical Oncology, Lyon University Hospital, Lyon, France) [olivier.glehen@chu-lyon.fr](mailto:olivier.glehen@chu-lyon.fr)

Sylvie ISAAC (Department of Pathology, Lyon University Hospital, Lyon, France) [sylvie.isaac@chu-lyon.fr](mailto:sylvie.isaac@chu-lyon.fr)

Marc POCARD (Department of Digestive Surgery, Lariboisiere University Hospital, Paris, France) [marc.pocard@gmail.com](mailto:marc.pocard@gmail.com)

François QUENET (Department of Surgical Oncology, Montpellier Cancer Institute, Montpellier, France) [Francois.Quenet@icm.unicancer.fr](mailto:Francois.Quenet@icm.unicancer.fr)

**French Sarcoma Group (GSF-GETO)**

Philippe ANRACT (Department of Oncologic Orthopedic Surgery, Cochin University Hospital, Paris, France) [philippe.anract@cch.ap-hop-paris.fr](mailto:philippe.anract@cch.ap-hop-paris.fr)

Jacques-Olivier BAY (Cellular Therapy and Clinic Hematology Unit, Hôtel-Dieu University Hospital, Clermont-Ferrand, France) [jobay@chu-clermontferrand.fr](mailto:jobay@chu-clermontferrand.fr)

Jean-Yves BLAY (Department of Medical Oncology, Centre Leon Berard, Lyon, France) [jean-yves.blay@lyon.unicancer.fr](mailto:jean-yves.blay@lyon.unicancer.fr)

Sylvie BONVALOT (Department of Surgery, Curie Institute, Paris, France) [sylvie.bonvalot@curie.fr](mailto:sylvie.bonvalot@curie.fr)

Christine Chevreau (Department of Medical Oncology, Claudius Regaud Institute, Toulouse, France) [Chevreau.Christine@iuct-oncopole.fr](mailto:Chevreau.Christine@iuct-oncopole.fr)

Frederic CHIBON (Department of Molecular Pathology, Bergonie Institute, Bordeaux, France) [F.Chibon@bordeaux.unicancer.fr](mailto:F.Chibon@bordeaux.unicancer.fr)

Jean-Michel COINDRE (Department of Pathology, Bergonie Institute, Bordeaux, France) [j.coindre@bordeaux.unicancer.fr](mailto:j.coindre@bordeaux.unicancer.fr)

Olivier COLLARD (Department of Medical Oncology, Institut de Cancérologie de la Loire Lucien Neuwirth, St Priest En Jarez, France) [Olivier.collard@icloire.fr](mailto:Olivier.collard@icloire.fr)

Gonzague DE PINIEUX (Department of Pathology, Trousseau University Hospital, Tours, France) [depinieux@med.univ-tours.fr](mailto:depinieux@med.univ-tours.fr)

Martine DELANNES (Department of Radiation Therapy, Claudius Regaud Institute, Toulouse, France) [Delannes.Martine@iuct-oncopole.fr](mailto:Delannes.Martine@iuct-oncopole.fr)

Florence DUFFAUD (Department of Medical Oncology, La Timone University Hospital & Aix-Marseille University, Marseille, France) [fduffaud@mail.ap-hm.fr](mailto:fduffaud@mail.ap-hm.fr)

François GOLDWASSER (Department of Medical Oncology, Cochin University Hospital, Paris, France) [francois.goldwasser@cch.aphp.fr](mailto:francois.goldwasser@cch.aphp.fr)

François GOUIN (Department of Orthopedic Surgery, Nantes University Hospital, Nantes, France) [fgouin@chu-nantes.fr](mailto:fgouin@chu-nantes.fr)

Antoine ITALIANO (Department of Medical Oncology, Bergonie Institute, Bordeaux, France) [A.Italiano@bordeaux.unicancer.fr](mailto:A.Italiano@bordeaux.unicancer.fr)

Axel LE CESNE (Department of Medical Oncology, Gustave Roussy, Villejuif, France) [Axel.LECESNE@gustaveroussy.fr](mailto:Axel.LECESNE@gustaveroussy.fr)

Perrine MAREC-BERARD (Department of Pediatric Oncology, Hematology and Pediatric Oncology Institute, Lyon, France), [perrine.marec-berard@ihope.fr](mailto:perrine.marec-berard@ihope.fr)

Eric MASCARD (Department of Pediatric Orthopedic Surgery, Necker Enfants Malades University Hospital, Paris, France) [eric.mascard@wanadoo.fr](mailto:eric.mascard@wanadoo.fr)

Simone MATHOULIN-PELISSIER (Clinical and Epidemiological Research Unit, Bergonie Institute, Bordeaux, France) [S.Mathoulin@bordeaux.unicancer.fr](mailto:S.Mathoulin@bordeaux.unicancer.fr)

Daniel ORBACH (Department of Adolescent and Pediatric Oncology, Curie Institute, Paris, France) [daniel.orbach@curie.fr](mailto:daniel.orbach@curie.fr)

Nicolas PENEL (Department of Medical Oncology, Centre Oscar Lambret, Lille, France), [n-penel@o-lambret.fr](mailto:n-penel@o-lambret.fr)

Sophie PIPERNO-NEUMANN (Department of Adolescent and Pediatric Oncology, Curie Institute, Paris, France) [sophie.piperno-neumann@curie.fr](mailto:sophie.piperno-neumann@curie.fr)

Françoise REDINI (UMR 957, Nantes University Hospital, Nantes, France) [francoise.redini@univ-nantes.fr](mailto:francoise.redini@univ-nantes.fr)

Philippe ROSSET (Department of Orthopedic Surgery, Trousseau University Hospital, Tours, France) [rosset@med.univ-tours.fr](mailto:rosset@med.univ-tours.fr)

Sophie TAÏEB (Department of Radiology, Centre Oscar Lambret, Lille, France) [s-taieb@o-lambret.fr](mailto:s-taieb@o-lambret.fr)

**French Pediatric Cancer Society (SFCE)**

Dominique VALTEAU-COUANET (Department of Pediatric and Adolescent Oncology, Gustave Roussy, Villejuif, France) [dominique.valteau-couanet@gustaveroussy.fr](mailto:dominique.valteau-couanet@gustaveroussy.fr)

Arnaud PETIT (Department of Pediatric Hematology and Oncology, Armand Trousseau University Hospital, Paris, France) [arnaud.petit@trs.aphp.fr](mailto:arnaud.petit@trs.aphp.fr)

Sabine IRTAN (Department of Pediatric Surgery, Armand Trousseau University Hospital, Paris, France) [sabine.irtan@trs.aphp.fr](mailto:sabine.irtan@trs.aphp.fr)

Herve BRISSE (Department of Radiology, Curie Institute, Paris, France) [herve.brisse@curie.net](mailto:herve.brisse@curie.net)

Jean-Hugues DALLE (Department of Hematology, Robert Debre University Hospital, Paris, France) [jhugues.dalle@gmail.com](mailto:jhugues.dalle@gmail.com)

Anne LAPRIE (Department of Radiation Therapy, Claudius Regaud Institute, Toulouse, France) [laprie.anne@iuct-oncopole.fr](mailto:laprie.anne@iuct-oncopole.fr)

Pierre LEBLOND (Pediatric oncology unit, Centre Oscar Lambret, Lille, France) [p-leblond@o-lambret.fr](mailto:p-leblond@o-lambret.fr)

Veronique MINARD-COLIN ((Department of Pediatric and Adolescent Oncology, Gustave Roussy, Villejuif, France) [Veronique.minard-colin@gustaveroussy.fr](mailto:Veronique.minard-colin@gustaveroussy.fr)

Isabelle PELLIER (Department of Pediatrics, Angers University Hospital, Angers, France) [ispellier@chu-angers.fr](mailto:ispellier@chu-angers.fr)

Françoise REDINI (UMR 957, Nantes University Hospital, Nantes, France) [francoise.redini@univ-nantes.fr](mailto:francoise.redini@univ-nantes.fr)
